# A note on Youden's *J *and its cost ratio

**DOI:** 10.1186/1471-2288-10-89

**Published:** 2010-09-30

**Authors:** Niels Smits

**Affiliations:** 1 Department of Clinical Psychology, Faculty of Psychology and Education, Vrije Universiteit, Van der Boechorststraat 1, 1081 BT Amsterdam, the Netherlands

## Abstract

**Background:**

The Youden index, the sum of sensitivity and specificity minus one, is an index used for setting optimal thresholds on medical tests.

**Discussion:**

When using this index, one implicitly uses decision theory with a ratio of misclassification costs which is equal to one minus the prevalence proportion of the disease. It is doubtful whether this cost ratio truly represents the decision maker's preferences. Moreover, in populations with a different prevalence, a selected threshold is optimal with reference to a different cost ratio.

**Summary:**

The Youden index is not a truly optimal decision rule for setting thresholds because its cost ratio varies with prevalence. Researchers should look into their cost ratio and employ it in a decision theoretic framework to obtain genuinely optimal thresholds.

## Background

In the clinical field there is a need for obtaining optimal cut offs on markers or tests for separating persons with a specific condition (diseased) from those without this condition (healthy). The quality of a clinical test with threshold *c *is often expressed in a table such as Additional file 1: Table S1.

In this table, *D *is the diagnosis; a person is either diseased (*D*+) or healthy (*D*-). The prevalence *P *is the proportion of persons diseased. *T *is the test result; *c *is a cut-off point on the test. Persons with test scores larger than or equal to *c *are tested positive and persons scoring below *c *are tested negative. Unfortunately, one is commonly faced with an imperfect relation between diagnosis and test result, and therefore there are four possible outcomes: false positives, true negatives, true positives, and false negatives. The abbreviations (FP*_c_*, TN*_c_*, TP*_c_*, and FN*_c_*) in Additional file 1: Table S1 represent the proportion of the population in each cell. When *c *changes, these proportions, and the level of the test *Q_c _*change as well. The quality of a medical test is often expressed in terms of the two conditional probabilities describing its performance with reference to the diagnosis. Sensitivity (SE) is the probability that a diseased person is tested as such (see, bottom of the table). Specificity (SP) is the probability that a healthy person has a negative test outcome. In general, SE and SP are inversely related and vary with the threshold: when using a higher (lower) value for *c*, SE*_c _*will decrease (increase) and SP*_c _*will increase (decrease).

An index for setting thresholds on tests is the Youden index [1], which is defined as

(1) $$J_{c}={\text{SE}}_{c}+{\text{SP}}_{c}-1.$$

The index is calculated for each threshold *c*, and the value c*, which achieves a maximum, is referred to as the 'optimal' threshold.

In the original article by Youden it was stated that the index "assumes false positives to be as undesirable as false negatives" ([1], p. 33), and that it "is independent of the relative sizes of the control and diseased groups" (Feature 5, p. 33). Although the paper was criticized at first [2], the index gained some popularity with clinical researchers (especially in psychiatry, see, e.g., [3]) and statisticians. Recently, for example, it was said to be the index which is "easiest to apply and does not require further information such as prevalence rates and decision error costs" ([4], p. 459), and that other indices are "influenced by the disease prevalence", whereas Youden's *J *is not ([5], par. 1.2.3).

All this seems to suggest that when using the index, i) incorrect classifications of healthy and diseased persons are equally costly, and ii) that prevalence does not play a role. As we will see, the former is only true in one specific situation. Moreover, the index is optimal with respect to misclassification costs which are related to the prevalence.

## Discussion

When determining optimal thresholds in a decision theoretic approach, an obvious first step is to calculate Expected Costs (EC) for each threshold, which can be directly obtained by multiplying the costs (C) of erroneous decisions and benefits (B) of correct decisions by their matching proportions and adding the four products (see, Additional file 1: Table S1):

(2) $$\begin{array}{r} {\text{EC}}_{c}=C_{FP}\times {\text{FP}}_{c}+C_{FN}\times {\text{FN}}_{c}\\ -B_{TN}\times {\text{TN}}_{c}-B_{TP}\times {\text{TP}}_{c}. \end{array}$$

The threshold with the lowest EC is the optimal threshold c*. Instead of looking at the benefits or costs of each of the four outcomes, it is often more convenient to consider the following ratio of costs (see, e.g., [6], p. 119):

(3) $$r=\frac{B_{TP}-C_{FN}}{B_{TP}+B_{TN}-C_{FN}-C_{FP}},$$

where *B_TP _*− *C_FN _*reflects how much difference in costs it makes whether diseased persons are classified correctly or not, and *B_TN _*− *C_FP _*reflects how much difference it makes whether healthy persons are classified correctly or not. The value of *r *will be close to one if the difference in costs between correct and incorrect classifications is much larger for diseased persons; if it is close to zero then this difference is much larger for healthy persons. Only when *r *is close to 0.5, the difference in costs is about equal for healthy and diseased persons. EC is often rewritten in terms of *r *and rescaled to improve its usefulness. For example, when rewriting Equation 2, and removing some constants [[7], Equation 3] we get EC':

(4) $${{\text{EC}}^{\prime }}_{c}=P\times {\text{SE}}_{c}\times r+\left(1-P\right)\times {\text{SP}}_{c}\times \left(1-r\right)$$

(5) $$={\text{TP}}_{c}\times r+{\text{TN}}_{c}+\left(1-r\right).$$

EC and EC' give identical c*.^1 ^For linking Youden's *J *to it, a further elaboration on the decision theoretic approach is not necessary. Kraemer ([6], pp. 121-123) showed, however, how EC, like SE and SP, is an uncalibrated measure in the sense that it depends on the level of the test and prevalence. She rescaled EC into index *κ*(*r*), which may be written as:

(6) $$\kappa {\left(r\right)}_{c}=\frac{{\text{TN}}_{c}\times {\text{TP}}_{c}-{\text{FN}}_{c}\times {\text{FP}}_{c}}{r\times P\times \left(1-Q_{c}\right)+\left(1-r\right)\times \left(1-P\right)\times Q_{c}}.$$

The index *κ*(*r*) is a weighted kappa coefficient [8] between test and diagnosis, and should range in value between zero and one. The optimal cut-off c* for a particular value of *r *is the one with the highest value of *κ*(*r*); EC and *κ*(*r*) do not necessarily give identical c*.

It is now informative to rewrite Equation 1. Given that subtracting a constant does not change the choice for c*,

(7) $${J^{\prime }}_{c}=\frac{{\text{TP}}_{c}}{P}+\frac{{\text{TN}}_{c}}{\left(1-P\right)}.$$

This can be reduced to:

(8) $${J^{\prime }}_{c}=\frac{{\text{TP}}_{c}\times \left(1-P\right)+{\text{TN}}_{c}\times P}{P\times \left(1-P\right)}.$$

Multiplying by a constant does not change the choice for c*:

(9) $${J^{\prime \prime }}_{c}={\text{TP}}_{c}\times \left(1-P\right)+{\text{TN}}_{c}\times P.$$

It can be easily seen that Equation 5 is equal to Equation 9 when *r *= (1 - *P*).^2 ^Consequently, Youden's *J *can be interpreted as a decision theoretic approach which uses a cost ratio equal to the proportion of healthy persons in the population (also see, e.g., [9], p. 572, or [10], p. 8). Thus, if the majority of the target population is diseased (1 - *P *= *r *< 0.5), classification errors with respect to healthy individuals are valued as more costly; if prevalence is low (1 - *P *= *r *> 0.5), classification errors with respect to diseased individuals are valued as more costly. Only when the prevalence is 0.5, does Youden's *J *evaluate classification errors for both groups as equally costly.

Index *J *is now contrasted with a comprehensive decision theoretical approach in which a fixed cost ratio is used. Additional file 2: Table S2 and Additional file 3: Table S3 tabulate the fictitious outcomes of a marker or test under three ordered thresholds (*c *equals 1, 2, and 3, respectively) for a group of 120 individuals. Additional file 2: Table S2 has a prevalence of 25%, and Additional file 3: Table S3 of 75%. Although the tables are different in the cells of the respective classification tables, they have identical sensitivity and specificity.

In this example it is assumed that a decision maker (that is, a given patient, physician, or health care system), has determined a fixed cost ratio expressing equal costs for false positives and false negatives, which comes down to *r *= 0.5. Although in practice this cost-ratio may be open to debate, we assume that it is a valid representation of the decision maker's costs and benefits. The last column of Additional file 2: Table S2 and Additional file 3: Table S3 show the weighted kappa under this cost ratio. In the 25% prevalence scenario, *κ*(0.5) is highest for *c *= 3. In the case of a prevalence of 75%, however, as a result of a different compound of correct and incorrect decisions in the classification tables, a different threshold, viz. *c *= 2, should be chosen.

By contrast, Youden's *J *(*κ*[1 - *P*]) gives identical values in both tables (see fifth column). The difference in compound of correct and incorrect decisions in like tables does not affect the choice of threshold: *c *= 2 is chosen in both. Obviously, different cost ratio's are used in both tables. In Additional file 2: Table S2, *r *= 1 - *P *= 0.75, which denotes that a misclassification of one diseased person is valued as equally costly as three misclassified healthy persons. By contrast, in Additional file 3: Table S3, a cost ratio *r *= 1 - *P *= 0.25 is used, which denotes that a misclassification of one healthy person is three times as costly as a misclassified diseased person.

It was shown that when using Youden's index to obtain optimal thresholds, one implicitly uses decision theory with misclassification costs which depend on the prevalence of the disease; more specifically, a cost ratio (costs for the diseased relative to the total costs) which is equal to one minus the prevalence proportion is employed. In addition, it was illustrated that in populations with identical test sensitivity and specificity, but with different prevalence, the employed cost ratio of *J *changes with the prevalence, whereas in a decision theory framework it is fixed. All this showed that, although it seems as if one can *choose *to take a decision theoretic approach (see, e.g., [11], p. 298) or not, the *use *of a chosen cut-off is invariably *optimal *with reference to some fixed cost ratio. Self-evidently, when using the Youden index, it is doubtful whether this population-dependent cost ratio actually represents the decision maker's preferences. Likewise, when using the index in populations with a different prevalence, the cut-off is optimal with reference to a different cost ratio. This may be undesirable, because it is highly unlikely that a decision maker's evaluation of classification errors varies from one population to another. For example, it is hard to imagine that an oncologist evaluates false negatives differently for males (higher prevalence) and females (lower prevalence) in testing for lung cancer.

It should be noted that there are settings, such as a health care system, in which the use of Youden's *J *makes sense. In a low prevalence population a health care system would typically use a screening test, and in such situations a desirable test would have high sensitivity and high predictive value of a negative test, since the consequence of a positive screening would be no more than a direction to see a doctor. The threshold with maximal *J *= *κ*(1 - *P*), *P *near zero would be optimal. In a high prevalence population, the health care system would prefer a test with high specificity and high predictive value of a positive classification, since a positive outcome would lead to clinical action such as invasive procedures. The threshold with maximal *J *= *κ*(1 - *P*), *P *near one would be optimal. This note, therefore, does not warn against using Youden's *J *per se, but against using it as a 'by default' method.

Sometimes, the consequences of incorrect test results and the disease prevalence may not yet have been determined, for example, in the early stages of test development. One may ask how an optimal cut-off should then be established. The answer is that in this phase there is no need for a cut-off yet. Instead, the main aim is to assess the *predictive utility *of the test. To that end an index such as the area under the curve of the receiver operating curve [9] may be used.

In practice, a precise determination of the costs and benefits of incorrect and correct classifications may be rather difficult. In such cases, attention may be restricted to *r*, the ratio of relative costs. Fortunately, the exact cost ratio is not needed in a decision theoretic framework; all that is needed is a qualitative indication of which of the two classification errors is more important to avoid [12]. For an illustration of determining the costs of misclassification in testing in psychiatry, see Smits et al. [7].

Although the Youden index is popular in some clinical domains, the most popular index for setting thresholds on medical tests is the odds ratio (see, e.g., [13]).^3 ^Kraemer [6,13] has shown that this index is unsuitable for this task, however. To illustrate why, the log transformation of this index is presented: log(odds ratio) = log(TN*_c_*) + log(TP*_c_*) - log(FN*_c_*) - log(FP*_c_*); commonly, the threshold with the highest log odds ratio is chosen. This formula shows that the logs of the two correct classification proportions get weight 1 and the logs of the two incorrect classification proportions get weight -1. Consequently, the two correct classifications on the one hand and two incorrect classifications on the other are lumped together, and a specific value of the log odds ratio may be the result of many different compilations of proportions in the classification table. Thus, the index tends to indicate whatever is the best quality of a test; for some tests this is sensitivity, and for other tests this is specificity [13]. For a decision theoretic approach, it should be known what quality is being optimized, and obviously the odds ratio does not provide this information.

Instead of automatically using Youden's index or the odds ratio, test developers should explicitly make use of decision theory for setting thresholds. To that end an abundant literature on setting genuinely optimal cut-offs, such as the book by Kraemer [6], is available.

## Summary

Youden's index, the sum of sensitivity and specificity minus one, is a method for obtaining thresholds on medical tests. It implicitly employs a ratio of misclassification costs which is equal to one minus the prevalence proportion. It is doubtful whether this cost ratio represents the decision maker's true preferences in all cases. In addition, from a decision theoretic point of view, the obtained threshold is optimal with reference to a variable cost ratio; when the test is used a in different population, the chosen threshold may be optimal with reference to a different cost ratio. It is argued that instead of using the index by default, researchers should explicate their cost ratio in a decision theoretic framework to obtain genuinely optimal thresholds.

## Competing interests

The author declares that he has no competing interests.

## Appendix

^1^Maximizing Equations 2 and 4 is equivalent to finding that particular cut-off point of the receiver operating characteristic curve where its slope equals (1 - *P *) × (1 - *r*)**/**(*P *× *r*) (e.g., [14], Eq. 1).

^2^It can also be shown that when using this cost ratio, *J *and *κ*(*r*) give identical results (also see, [13], Table II). Noting that TP*_c_*= SE*_c _*× *P*, TN*_c _*= SP*_c _*× (1 - *P*), FN*_c _*= (1 - SE*_c_*) × (1 - *P*), and FP*_c _*= (1 - *SP*) × *P *, the numerator of Equation 6 can be written as *P *× (1 - *P *) × SE*_c _*× SP*_c _*- (1 - SE*_c_*) × (1 - SP*_c_*) = *P *× (1 - *P*) × (SP*_c _*+ SE*_c _*- 1). The denominator can be rewritten as *r *× *P *+ *Q_c _*× (1 - *r *- *P*). This leads to the following equation, *κ*(*r*)*_c _*= *P *× (1 - *P *) × (SP*_c _*+ SE*_c _*- 1)**/**(*r *× *P *+ *Q_c _*× [1 - *r *- *P*]). It can be easily seen that Equation 6 reduces to Equation 1 when *r *= 1 - *P*.

^3^Warrens [15] showed that the odds ratio can be transformed into a weighted kappa. These two indices do not necessarily lead to identical choices for *c*, however.

## Pre-publication history

The pre-publication history for this paper can be accessed here:

http://www.biomedcentral.com/1471-2288/10/89/prepub

## Supplementary Material

Additional file 1 **Table 1 - Decision table for testing situation**. This is the decision table with some squares and some definitions in the middle. Click here for file

Additional file 2 **Table 2 - Situation 1, prevalence is 25%**. This contains several two by two tables in a large table, the entries in the first small table are 36, 54, 6, and 24. Click here for file

Additional file 3 **Table 3 - Situation 2, prevalence is 75%**. This contains several two by two tables in another large table, the entries in the first small table are 12, 18, 18, and 72. Click here for file
